# Archaeal and bacterial communities across a chronosequence of drained lake basins in arctic alaska

**DOI:** 10.1038/srep18165

**Published:** 2015-12-18

**Authors:** J. Kao-Kniffin, B.J. Woodcroft, S.M. Carver, J.G. Bockheim, J. Handelsman, G.W. Tyson, K.M. Hinkel, C.W. Mueller

**Affiliations:** 1 Cornell University, School of Integrative Plant Science, Ithaca, NY 14853, United States; 2 University of Queensland, Australian Centre for Ecogenomics, School of Chemistry and Molecular Biosciences, Brisbane 4072, Queensland, Australia; 3 University of Wisconsin-Madison, Department of Soil Science, Madison, WI 53706, United States; 4 Yale University, Department of Molecular, Cellular, and Developmental Biology, New Haven, CT 06520, United States; 5 University of Cincinnati, Department of Geography, Cincinnati, Ohio, 45221, United States; 6 Technische Universitaet Muenchen (TUM), Lehrstuhl fuer Bodenkunde, 85354 Freising, Germany

## Abstract

We examined patterns in soil microbial community composition across a successional gradient of drained lake basins in the Arctic Coastal Plain. Analysis of 16S rRNA gene sequences revealed that methanogens closely related to *Candidatus* ‘Methanoflorens stordalenmirensis’ were the dominant archaea, comprising >50% of the total archaea at most sites, with particularly high levels in the oldest basins and in the top 57 cm of soil (active and transition layers). Bacterial community composition was more diverse, with lineages from OP11, Actinobacteria, Bacteroidetes, and Proteobacteria found in high relative abundance across all sites. Notably, microbial composition appeared to converge in the active layer, but transition and permafrost layer communities across the sites were significantly different to one another. Microbial biomass using fatty acid-based analysis indicated that the youngest basins had increased abundances of gram-positive bacteria and saprotrophic fungi at higher soil organic carbon levels, while the oldest basins displayed an increase in only the gram-positive bacteria. While this study showed differences in microbial populations across the sites relevant to basin age, the dominance of *Candidatus* ‘M. stordalenmirensis’ across the chronosequence indicates the potential for changes in local carbon cycling, depending on how these methanogens and associated microbial communities respond to warming temperatures.

The Arctic wetlands of the northern Coastal Plain of Alaska hold vast reserves of carbon (C) that play an important role in global climate regulation^1^. A synthesis of continuous permafrost ecosystems in the late 20^th^ century shows that colder permafrost sites are warming more rapidly^2^. Just over the last hundred years, the mean air temperature in the Arctic Coastal Plain of Alaska rose 2 to 6 °C^3^. Moreover, warmer temperatures are predicted to enhance the decomposition of both labile and non-labile soil organic carbon (SOC)^4^,^5^. Temperature-sensitive microbial decomposition of SOC stimulates the release of carbon dioxide (CO_2_) and methane (CH_4_) from soil, which could lead to a positive feedback resulting in increasingly warmer temperatures^6^,^7^. While temperature-dependent physiological responses contribute to the feedback scenario, changes in the structure of the microbial community associated with accelerated warming or permafrost decline could be useful as predictive variables in future CO_2_ and CH_4_ emissions.

The Arctic Coastal Plain of Alaska provides a heterogeneous landscape to characterize soil microbial community structure relevant to climate change. Nearly 75% of the Barrow Peninsula of northern Alaska is covered with lakes and drained lake basins differing in formation age (Fig. 1, Table 1)^8^ and soil C characteristics. Large amounts of SOC are present at the site, of which 25 kg SOC m^−2^ are stored as easily degradable organic matter rich in carbohydrates, and only 10 kg OSC m^−2^ are present as more stable, mineral-associated organic C^9^. Although much of the C is stored securely in permafrost, continued warming trends could perturb the formation and drainage of thaw lakes that follow a 5,000 year cycle of successional development^10^, with consequences on C cycling. For example, CO_2_ emissions may increase through enhanced microbial decomposition of labile and non-labile SOC that was once inaccessible in permafrost.

Understanding SOC dynamics in relation to soil microbial community composition^11^ could provide clues on CO_2_ and CH_4_ emissions when the upper permafrost thaws. A global meta-analysis of published data revealed a strong correlation between microbial biomass and soil C availability^12^. Similarly, a survey of 400 soil samples around the world confirmed that the variation in microbial biomass across biomes was most strongly related to SOC levels^13^. In this study, we examined soil microbial community composition across a chronosequence of drained lake basins, including young (<50 yr), medium (<300 yr), old (<3,000 yr), and ancient (3,000–5,000 yr) basins (Fig. 1 and Supplementary Figure 1) at three depth layers (active, transition, and permafrost). Thaw lake development is comprised of distinct vegetation communities^14^ that contribute to patterns in SOC, such that the organic layer of the basins thickens with greater accumulation of plant matter^15^.

The heterogeneity of the drained lake basin landscape suggests that C cycling dynamics may be distinct across the chronosequence. The composition of SOC across the chronosequence is distinct between young basin soils versus the medium, old, and ancient basins, with a large amount of SOC in the form of particulate organic matter at depth in these older basins^9^. The differences in SOC characteristics between the young and older basins, could be influencing soil microbial composition and function, and determining respiration of CO_2_ and methane. Correspondingly, a study conducted in the same area showed that the youngest drained lake basins had the highest levels of ecosystem respiration^16^. If basins show distinct microbial community composition and biomass across the chronosequence, the ability to predict microbial responses to environmental change could be improved. Specifically, we hypothesized that: 1) microbial diversity will increase across the chronosequence, reflecting the shift toward more bioavailability of particulate organic matter found in older basins; and 2) the abundance of specific microbial groups will be related to soil C levels.

## Results

### Archaeal population composition

The majority of the archaeal OTUs belonged to the phylum Euryarchaeota, including members of methanogenic genera *Methanobacteria*, *Methanoregula*, *Methanosarcina*, and *Methanosaeta*. The presence of lineages capable of using acetoclastic and/or hydrogenotrophic pathways indicates that a range of substrates can be used to generate methane in these environments (Fig. 2a). Redundancy analysis of the archaeal populations at each site showed that their relative abundance was significantly correlated with soil layer (p < 0.005) and basin age (p < 0.046; after accounting for soil layer).

Methanogens belonging to *Candidatus* ‘Methanoflorens’ were found in all samples, and were the dominant archaea in most communities (>50% of archaeal sequences in most basins) (Fig. 2a). The ‘Methanoflorens’ OTU was 99% identical to *Candidatus* ‘Methanoflorens stordalenmirensis’, recently discovered in thawing permafrost at Stordalen Mire, Sweden^17^. Although the ‘Methanoflorens’ OTU was found in basins of all ages, it was present at higher abundance in the active and transition layers (Fig. 3). Both ‘Methanoflorens’ and other methanogenic lineages were also found in the permafrost, albeit at a lower relative abundance.

Members of the anaerobic methanotrophic clade ANME-2, including *Candidatus* family ANME-2d (recently named Methanoperedenaceae)^18^ were detected in many basins. However, ANME 2a-2b appeared in only one basin. At a few sites, the ANME-2 were found in high abundance (up to 64%), primarily in the permafrost layer (Fig. 2a).

Other archaeal members included the Miscellaneous Crenarchaeota Group (MCG) and Parvarchaea. The OTUs belonging to the MCG class were evenly and consistently abundant across all basins and depths, but at a low to moderate relative abundance (1–24% of archaea, mean 13%). The Parvarchaea, although found at similar relative abundances, were found in only half of the basins sampled.

### Bacterial population composition

The bacterial populations were dominated by members of the Actinobacteria, Bacteroidetes, OP11 and Proteobacteria phyla (Fig. 2b). Similar to the archaeal populations, the variation in relative abundance of bacterial populations was significantly correlated with soil layer (p-value <0.005). However, basin age did not significantly influence bacterial relative abundance. Instead, soil bulk density was found to be a better predictor (p-value <0.005; after accounting for soil layer).

Other bacterial taxa found at the site were not present in all basins, but appeared in most. An OTU in the actinobacterial family Intrasporangiaceae was more prevalent in transition and permafrost layers (up to 32% of Bacteria), and abundant in medium, old, and ancient basins. Members of the order Bacteroidales (Bacteroidetes) and the class OP11-2 (OP11) were abundant (up to 34%), particularly in the active layer. An OTU belonging to the betaproteobacterial genus *Rhodoferax* was present across all soil layers. A gammaproteobacterial OTU belonging to the Aeromonadales increased in relative abundance and prevalence in the transition and permafrost layer samples. Eight other phylogenetically novel OTUs (89–95% sequence identity to the closest hit in GreenGenes) were also observed sporadically at high abundance. These lineages were related to members of the families Deinococcaceae and Anaerolineae, and the orders OP11-4 and WCHB1-64 within the OP11^19^.

### Comparison of microbial communities across the chronosequence

Comparison of microbial communities across all sites and depths was undertaken by examining the beta diversities between all pairs of sites, as measured by unweighted Soergel (unweighted UniFrac) distance (Fig. 4). This analysis indicates that in general, active layers share a distinct community structure that differentiates them from transition and permafrost samples. Transition layer communities were moderately similar to each other (0.54 ± 0.04) and to both active layer (0.57 ± 0.06) and permafrost communities (0.57 ± 0.06). Wilcox rank sum tests also suggest lower beta diversity within active layer samples (p-values 0.005 and 10^−15^ relative than within transition and within permafrost layer beta diversities respectively, though not all beta diversity measures are independent observations). Permafrost communities were more highly differentiated, neither similar to other permafrost communities (0.60 ± 0.06) or active layers (0.61 ± 0.07). The highly similar community structure of the active layer samples was also observed in a principal coordinates analysis (PCoA) using beta diversities as distances (pairwise unweighted UniFrac distances average ±  standard deviation 0.52 ± 0.08) (Fig. 5). The active layer samples cluster together and away from the transition and permafrost. There is considerable overlap between transition and permafrost samples.

### Microbial biomass and soil depth

Microbial biomass differed across soil depth (Fig. 6), but showed no differences across basin age (data not shown). Microbial biomass in the active layer of soil was 1.6-fold higher than in the permafrost layer (F = 1.51, p = 0.01). The total biomass at the active versus permafrost soil layers averaged 327 and 206 nmol PLFA · g^−1^ soil, respectively, while microbial biomass in the transition layer averaged 265 nmol PLFA · g^−1^ soil. The pattern of decreasing microbial biomass with soil depth was consistent across all age basins. The relative abundances (or proportions) of major lipid indicators, such as monounsaturated (gram-negative bacteria), branched-chain (gram-positive bacteria), and fungal, did not differ across the basin chronosequence or with soil depth.

The environmental properties analyzed indicated patterns across basin age and depth, but no significant interactions between age and depth (Table 2). Soil pH was lower in the old basins compared to the young basins, while the medium and ancient basins showed no differences in pH level (F = 3.8, p < 0.05). Soil bulk density and SOC were also lower in older basins compared to the young basins (F = 4.9, p < 0.01 and F = 3.1, p < 0.05, respectively). Total nitrogen, soil C:N ratios, and soil moisture showed no patterns across the age basins. We found that the storage of C in the basins did not differ across the basin chronosequence, despite the large differences in organic layer thickness and carbon quality reported in a previous study^15^. Instead soil C stock differed only across depth, with the active layer soils containing the highest levels of stored C (F = 3.3, p < 0.05) at a soil depth of up to 100 cm.

PLFA indicated that gram-positive bacterial biomass was positively correlated with the concentrations of SOC for the young and ancient basins (Fig. 7a), while only the young basins showed a positive relationship with saprotrophic fungi (Fig. 7b). There were no correlations between microbial biomass and SOC in the medium and old basins. The soil properties measured showed no patterns with other indicator groups, such as the gram-negative bacteria, mycorrhizal fungi, and actinomycetes.

## Discussion

Accelerated warming has been more pronounced in the Arctic in recent decades, and given the continued warming trend, regions of thaw in permafrost landscapes provide a glimpse of the changing composition of permafrost microbial communities. The chronosequence of drained lake basins near Barrow, Alaska provides a landscape pattern representative of the Arctic Coastal Plain of Alaska, where basins span across a developmental range of 5,000 years. Each summer, the uppermost zone of soil, termed the active layer, thaws and provides a distinct zone to examine microbial community composition relative to deeper, continuously frozen soils (transition and permafrost layers). We found that ‘Methanoflorens’ are the dominant archaea in this landscape, with highest abundances in the active layer (>50% abundance in more than half of the samples). Their presence at significant levels in transition and permafrost layers indicates that methanogenesis may be possible within permafrost. Methanogenic activity in permafrost has previously been confirmed in the Siberian Arctic at temperatures from −3 to −6 °C^20^. Given the high relative abundance of ‘Methanoflorens’ observed at Stordalen Mire, Sweden, its presence within the Alaskan permafrost further supports the hypothesis that it may be a significant early methanogenic responder to permafrost thaw globally^21^.

Other archaea found consistently across the basins include members of crenarchaeal class MCG. Previous work in the Canadian High Arctic, using 16S rRNA gene clone libraries, showed that the archaeal community in the active and permafrost layers was comprised predominately of Crenarchaeota^22^. This estimation contrasts the levels we found using pyrosequencing, with a mean of only 13% relative abundance in the basins. The MCG are widespread and abundant in marine sediments^23^ and a variety of lakes^24^, but the lack of cultivable references makes it challenging to understand the ecology of these organisms. As MCG are neither methanogens nor methanotrophs, their contribution to C dynamics is unknown beyond heterotrophy.

Notable archaea that were found at the site, but infrequent or partial in occurrence, include members of the ANME lineage and the Parvarchaea. Sequences belonging to two ANME lineages (ANME-2a/b and *Candidatus* Methanoperedenaceae) were detected in several samples, and are reported to be found in freshwater or marine environments^25^. Other ANME clades have been detected in terrestrial ecosystems^26^. Members of the ANME-2a/b form aggregates with sulfate reducers in order to couple anaerobic oxidation of methane with sulfate reduction^27^. Consistent with the occurrence of ANME-2a/b, the OTU belonging to the family Desulfobulbaceae were also detected in several samples. A member of the ‘Methanoperedenaceae’ (ANME-2d) was recently shown to perform anaerobic methane oxidation coupled to nitrate reduction^18^. The co-occurrence of methanogens and methanotrophs in the same environment suggests that methane may be both produced and consumed simultaneously in some of these sites. Methanogens and anaerobic methanotrophs are also typically co-habitants of deep sea hydrothermal vents; however in this environment, ANME outnumber methanogens^28^,^29^. Even lesser known are the Parvarchaea, which are considered an ultra-small size (<500 nm diameter). They have previously been found in a Californian mine^30^ and in a Volcanic Lake in Costa Rica^31^. To our knowledge, this is the first report of Parvarchaea in a permafrost environment, which makes its role in Arctic biogeochemistry unknown.

Unlike the archaea, in which a dominant taxa was found, the bacterial taxa at our site were more evenly spread across the basins and soil depth layers. The Actinobacteria and Bacteroidetes were the most abundant and widely dispersed taxa across the chronosequence, followed by OP11 and Proteobacteria–all are common in marine and terrestrial environments^32^,^33^,^34^. Noticeably absent in our samples were the Acidobacteria, which were highly abundant in other Arctic environment soils^22^,^23^,^35^. The Actinobacteria are gram-positive bacteria that are well represented with many cultivable members that produce a variety of bioactive compounds, and are well known for their abilities to degrade complex C compounds^32^. The large quantities of SOC found in the Arctic Coastal Plain may have enriched for bacteria capable of accessing nitrogen and phosphorus bound in organic matter. Also abundant in numbers were the Bacteroidales, which are gram-negative, non-spore-forming bacteria found in both aerobic and anaerobic environments^36^. Altogether, members of the OP11 candidate divisions were distributed in most basins. They are found widely in a diversity of environments that include microbial mats, lakes, landfills, sulfur hot springs, termite guts, and other mostly anoxic environments^34^. Other bacterial taxa appeared infrequently across the chronosequence or soil depth layers, and often in low abundance. These taxa include the Firmicutes, Chloroflexi, and Deinococcus-Thermus. Low abundance of the Firmicutes was also found in Canadian High Arctic samples^33^. The poor representation of these taxa and the Acidobacteria across the chronosequence suggests that they may play a limited role in the ecology of the Arctic Coastal Plain sediments. It is important to note that estimations of higher abundance for certain taxa, including the Acidobacteria, could be erroneous as a result of widespread contamination in common DNA extraction kits and laboratory reagents^37^. This discrepancy in taxa abundance is particularly problematic in samples of low microbial biomass, such as in permafrost.

Microbial community composition in upper permafrost and transition layers provides a useful snapshot of relative abundances of key members, but it is unknown whether they are active or dormant cells, or preserved necromass. Within permafrost, the C sources used for methanogenesis are abundant and likely derived from the large pool of easily degradable, carbohydrate-rich SOC found at the site^9^. This labile pool of C can be redistributed from the active layer, where plant residues are abundant, to lower permafrost depths through cryoturbation^38^. Although it is conceivable that C is securely buried at greater depth with no active heterotrophy, subtle increments in Arctic soil temperature could result in rapid community changes and increased respiration of C in the permafrost^38^. Estimations of the number of dormant cells in the Arctic permafrost range from 0.02% in the Canadian High Arctic^39^ to 26% of the total cells in Spitsbergen, Norway^40^. These and other estimates^20^ indicate that some permafrost microbial communities are capable of utilizing C sources at low temperatures. Mineralization of ^14^C-labeled acetate and glucose at temperatures as low as −15 °C suggests that permafrost microbial communities are indeed capable of utilizing C buried at depth, and do not require thawing to revive cells for methanogenesis^41^,^42^.

While dormancy was not measured in this study, we assessed microbial biomass of major groups to shed additional light on community measurements not needing amplification methods. Microbial phospholipid fatty acids (PLFA) are useful markers for studying the effects of global change on living and recently living microorganisms. The PLFAs degrade rapidly in soils, in comparison to microbial amino sugars and DNA that can persist for decades or hundreds of years^43^. Microbial biomass levels in the deeper permafrost soils in our study were relatively high (206−265 nmol PLFA · g^−1^), and comparable to levels found in grassland and agricultural soils^44^,^45^. Surprisingly, active layer soils harbored only slightly more microbial biomass, 1.6-fold higher than permafrost (Fig. 6). This difference in biomass contrasts with previous findings where viable cell counts in the active layer are generally 100–1000 times greater than in the permafrost^46^,^47^. Microbial PLFAs are typically considered a way to estimate living microorganism abundance, because phospholipids are rapidly degraded in soils^43^. If only a fraction of the microbial biomass in permafrost at Barrow is active, it could have a significant contribution to C cycling in the Arctic Coastal Plain.

In active layer zones, where microorganisms are active during the summer months, microbial composition is similar across the basin chronosequence, whereas the deeper transition and permafrost layers harbor communities showing greater dissimilarity (Fig. 4). Differences in microbial community composition could be preserved in the frozen permafrost, but thawing could result in the convergence of the microbial communities to similar compositions^48^. Alternatively, the consistently low levels of similarity between permafrost communities relative to the active layer communities may indicate that microbial community structure in permafrost has evolved over time to be more specialized. Examining microbial communities in existing thaw zones thus provides insight into the biology of permafrost loss where microbial adaptation to temperature increases could result in predictable changes to microbial composition and activity^49^,^50^,^51^. Our observation that microbial communities are similar in thaw zones across the basin chronosequence, despite differences in soil parameters, suggests that temperature could be driving microbial composition in the Arctic Coastal Plain.

While soil C is considered a driver of microbial community composition^11^, we did not find a correlation with 16S rRNA gene data. Instead, increases in SOC were associated with greater biomass of gram-positive bacteria in young and ancient basins and saprotrophic fungi in young basins, as determined by PLFA (Fig. 7). These results contrast other studies showing that the relative abundance of gram-positive bacteria decreases with greater C addition^52^,^53^. It is tempting to conclude that the quality of C, as opposed to the quantity of SOC, may be a better determinant of microbial biomass. Cores analyzed from many of these same sites using NMR techniques indicate a large quantity of labile organic matter in older basins^9^. Furthermore, microbial respiration at the sites was not found to be limited by substrate availability^54^. Despite the high amounts of bioavailable C in older basins, the young basins may support greater microbial activity, as shown in an earlier study where ecosystem respiration levels were indistinguishable between the medium and old basins, but greater in the young basins^16^. Clearly, other parameters other than SOC quantity could be explaining microbial community composition in the Arctic Coastal Plain. Future studies focusing on more detailed associations between microbial communities and a multitude of SOC parameters are needed to understand how permafrost loss will impact regional C cycling.

## Methods

### Site description

**Figure 1:**
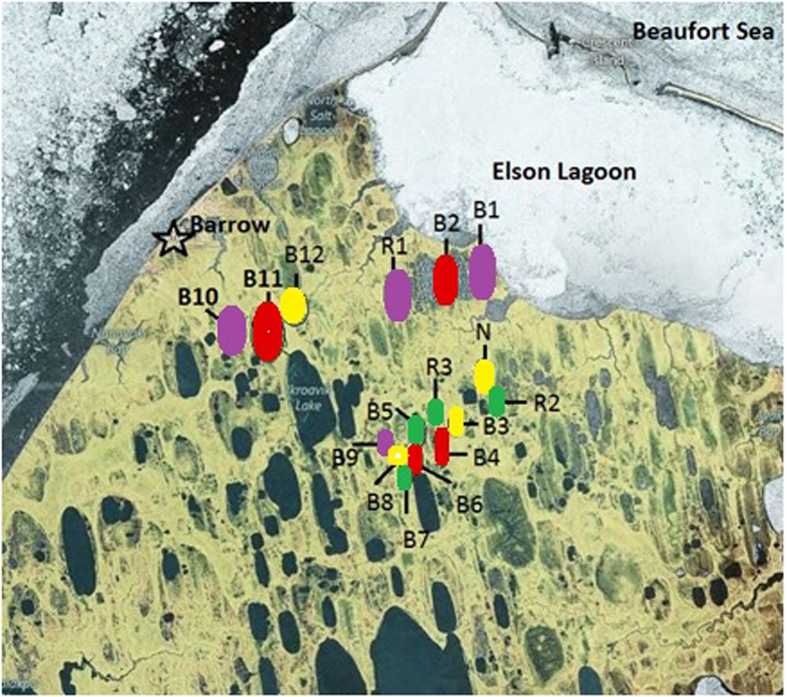
Location of sites that represent a chronosequence of drained lake basins. The sites range in age from <50 yr to <5,000 yr since drainage of lake waters. The basins used in this study are shown in color, and Barrow is identified with a star. Yellow represents young basins; purple represents medium basins; red represents old basins; and green represents ancient basins. Image generated using Bing Maps. Basin ages were previously classified by textural analysis of spectra from the Landsat imagery^74^ using 40 + age-dated drained lake basins as ground truth, including all 16 basins represented in the figure.

**Figure 2:**
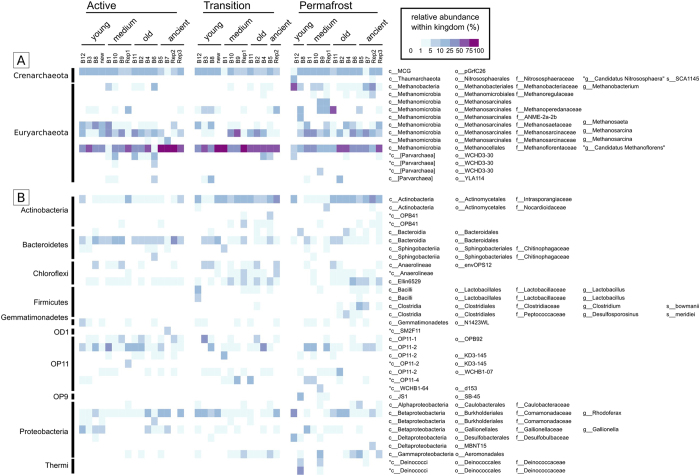
Relative abundances of archaea and bacteria. Heat maps of the relative abundances of (**a**) archaeal OTUs and (**b**) bacterial OTUs. Vertical columns show drained thaw lake basin sites (see Table 1 and Fig. 1 for basin ID) and horizontal rows represent OTUs. The color gradient on the lower right hand of each heat map shows increasing relative abundance with color intensity. Stars indicate that the best hit is <97% identical to the OTU’s representative sequence and the sequence found in GreenGenes 2012 and thus taxonomy is not confidently assigned. Deinococaceae OTUs have <95% identity to their best hit and so are only assigned to the family level.

**Figure 3:**
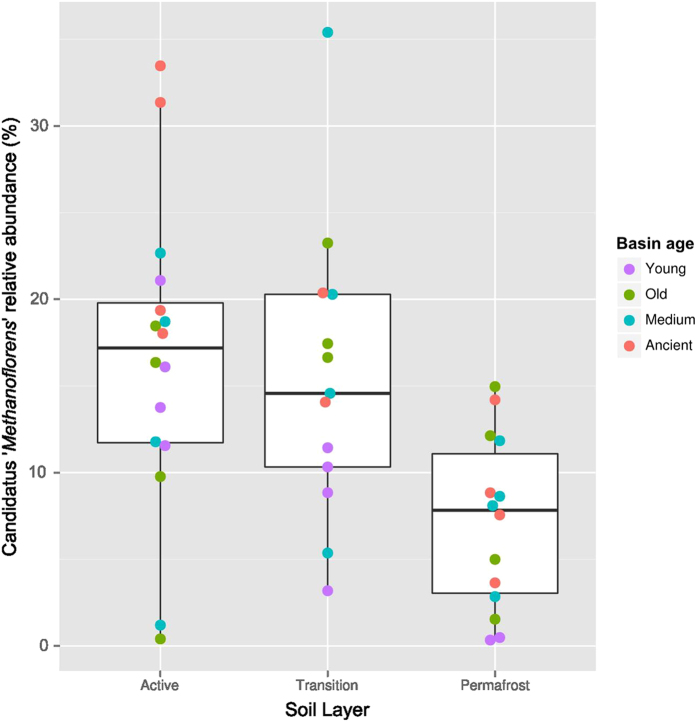
*Candidatus ‘*Methanoflorens stordalenmirensis” abundance. The relative abundance of the methanogen is plotted by percentage along soil depth layers. The following colors refer to the age category of the 16 basins: young (pink), medium (green), old (blue), and ancient (purple).

**Figure 4:**
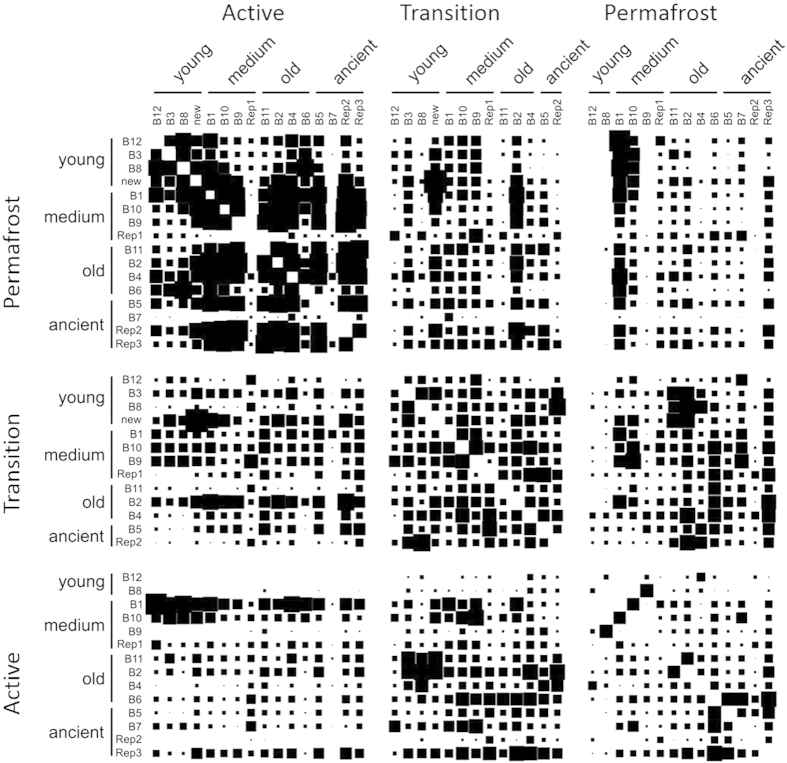
Pairwise beta diversity of total microbial communities. Each square represents the unweighted UniFrac distance between a pair of samples, where increasing square size indicates increased similarity (less beta diversity). A total of 16 basins (young, medium, old, and ancient) were sampled at three soil layer depths (active, transition, permafrost).

**Figure 5:**
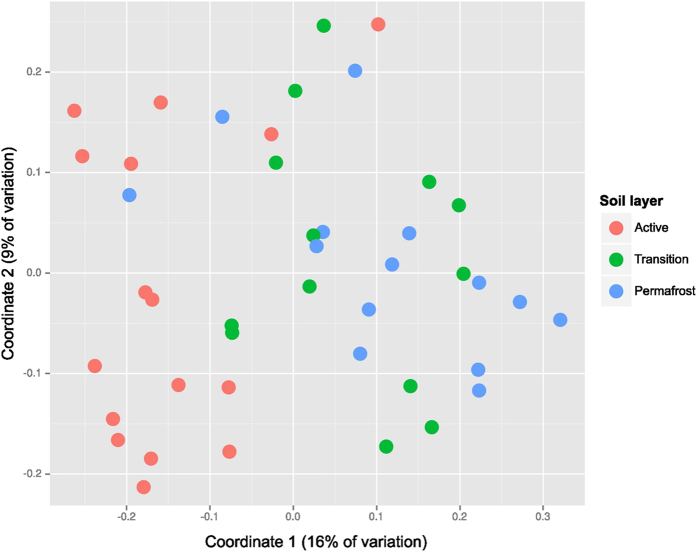
Convergence of microbial community composition in zones of thaw. Principal coordinates analysis (PCoA) using unweighted UniFrac as the distance measure between the total microbial communities. A total of 16 basins (young, medium, old, and ancient) were sampled at three soil layer depths: active (red), transition (green), and permafrost (blue).

**Figure 6:**
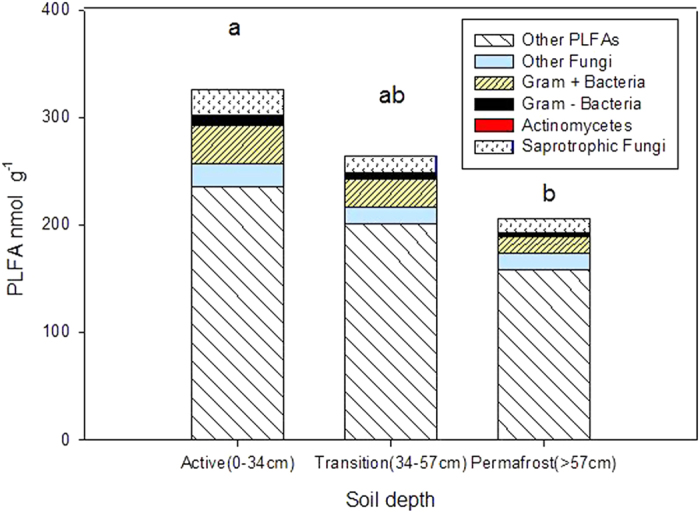
Soil microbial biomass across major microbial guilds by soil depth. Contrasting letters indicate significant differences in total lipid biomass means across the active, transition, and permafrost depth layers (Tukey’s HSD, p < 0.05). Means represent n = 46 basin samples.

**Figure 7:**
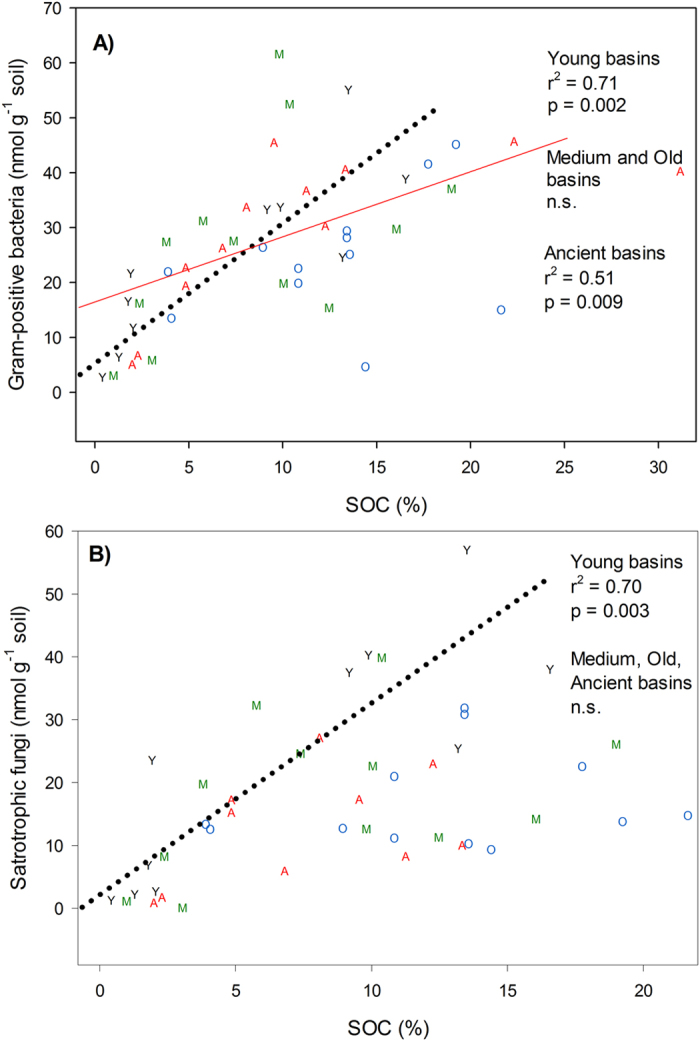
Patterns in microbial biomass and SOC. Microbial biomass (lipid nmol g^−1^ soil) in relation to SOC for: a) gram-positive bacteria and b) saprotrophic fungi. The young basins are plotted as Y in black color, medium basins are M in green color, old basins are O in blue color, and ancient basins are A in red color. Regressions indicating significance at p < 0.05 are shown with their respective r^2^ and p-values. The dashed black line represents the young basins and the solid red line corresponds with the ancient basins.

The Barrow Peninsula is located along the northern coast of Alaska and is part of the Arctic Coastal Plain. The sampling region is between 71°20′ to 71°27′ N latitude and between 156°4′ and 156°7′ W longitude. The mean annual air temperature ranges from 4 °C in the summer to −26 °C in mid-winter. The mean annual precipitation is 106 mm, with over 60% falling as rain from July through September. A total of 16 drained lake basins were sampled near Barrow, Alaska, from the following age classes: young (0–50 yr), medium (50–300 yr), old (300–2000 yr), and ancient (2000–5500 yr) (Fig. 1, Table 1). Identification and dating of the age classes were determined by using direct detection of ^14^C in samples with radiocarbon accelerator mass spectrometry^8^.

For each age class, four basins were examined for environmental parameters, including bulk density, soil pH, soil organic carbon (SOC), C:N ratio, total nitrogen (TN), C stock (kg · m^−2^), and soil microbial community composition. Soil moisture was similar across the chronosequence at the time of sampling (April 2010), where each core section was collected frozen and soils were fully saturated with water upon thaw. At each drained lake basin, a SIPRE corer measuring 80 to 150 cm long and 7.5 cm diameter was used to sample cores. The corer was attached to a Big Beaver earth drill apparatus (Little Beaver, Inc., Livingstone, TX) mounted on a sledge (Supplementary Figure 2). Within 8 hr, the frozen cores were taken to a cold room in Barrow and cut with a chop-saw into sections of corresponding soil horizons or depth-increments. The subsections of cores were frozen at −20 °C, packed with dry ice, and then shipped overnight to Madison, WI for further analyses.

### SSU rRNA gene amplicon analysis

DNA was extracted from thawed samples that were kept frozen at −80 °C using the PowerSoil DNA extraction kit (MoBio Laboratories, Carlsbad, CA), quantified, and checked for quality via PCR amplification. Three of the frozen soil samples were lyophilized (Active B12, and Permafrost R1 and R2). Active B12 sample initially yielded low DNA quantities and so was re-extracted with the following modifications: vortexing for 30 min and eluting with 50 μl of solution C6 rather than 100 μl. Permafrost R1 and Permafrost R2 samples were extracted with the PowerMax Soil DNA extraction kit due to low yield. The DNA concentration was determined using Quant-iT dsDNA assay kit (Invitrogen, Carlsbad, CA). Amplification was achieved with universal primers 926F (5′-**CCTATCCCCTGTGTGCCTTGGCAGTC**TCAGAAACTYAAAKGAATTGRCGG-3′) sequencing adapter in bold, key underlined and SSU-specific primer following) and 1392wR (5′-CCATCTCATCCCTGCGTGTCTCCGACTCAGXXXXXACGGGCGGTGWGTRC), where Xs indicate a variable length multiplex identifier listed in Supplementary Table 1 (similar to a tested primer set^55^ in Engelbrektson *et al.*, 2010). Template DNA (2.5 or 5 μl) was amplified in duplicate 50 μl reactions containing 1 U Taq DNA polymerase (Fisher), 0.2 mM dNTP mix (Fisher), 2 mM MgCl2 (Fisher), 2  μM of each primer and 10  μg μl−1 BSA (NEB). PCR was in a Veriti thermocycler (Applied Biosystems, Carlsbad, CA, USA) with an initial denaturation step of 95 °C for 3  min, 30 cycles of dissociation at 95 °C for 30 s, annealing at 55 °C for 45 s, extension at 74 °C for 30 s and final extension of 10 min at 74 °C. Sequencing was carried out on a Roche 454 GS-FLX at the Australian Centre for Ecogenomics. SSU rRNA gene sequences were processed using APP 3.0.3-3.0.4 (https://github.com/Ecogenomics/APP), with default parameters. Specifically, sequence files were demultiplexed using QIIME^56^, homoploymer errors corrected using Acacia 1.50^57^ and resulting reads were processed using the CD-HIT-OTU pipeline^58^. All reads were trimmed to 250 bp and reads <250 bp were discarded. OTUs were clustered at 97% nucleotide identity and representative sequences assigned taxonomy using BLASTN 2.2.22^59^ against the 2012 version of GreenGenes database^60^ (McDonald *et al.*, 2011). Operational taxonomic unit abundances were visualized with krona^61^. Alpha diversity measures including Simpson’s and Shannon’s index were calculated with express beta diversity^62^ (Supplementary Figure 4).

### Microbial lipid analysis

We used a modified method for PLFA analysis to characterize the composition of the soil microbial community. The resulting lipid biomarkers are separated into microbial guilds based on fatty acid structure. The method combines the first steps of PLFA sample extraction with the steps described in a more recent paper^63^. Briefly, the frozen soil samples were lyophilized and ground into fine particles prior to PLFA extraction. All glassware used in the extraction was baked at 550 °C for 3 hrs to remove fatty acid residues. Similarly, teflon tubes and caps were rinsed with hexane to remove residues prior to sample extraction. We extracted lipids from 3 g of lyophilized soil using a chloroform-methanol extraction with a phosphate buffer consisting of potassium phosphate (3.6 ml), methanol (8 ml), and CHCl_3_ (4 ml) in 25 ml glass tubes. The samples were further purified and processed through saponification and methanolysis using the FAME procedure described by Microbial ID Inc. (Hayward, CA).

The purified fatty acids (2 μl) were injected into a Hewlett-Packard 6890 Gas Chromatograph (GC) equipped with a flame ionization detector and split/splitless inlet. The GC was equipped with a 25 m × 0.2 mm inside diameter x 0.33 μm film thickness Ultra 2 (5%-phenyl, 95% methyl) capillary column (Agilent, Santa Clara, CA). Hydrogen was used as the carrier gas, N as the make-up gas, and air to support the flame. GC parameters were set by the MIDI Sherlock program (MIDI, Inc. Newark, DE). Peaks were identified with fatty acid standards and Sherlock peak identification software (MIDI, Inc. Newark, DE). Two internal standards, 9:0 (non-anoic methyl ester) and 19:0 (non-adeconoic methyl ester), of known concentration were used to create peak areas for calculation of corresponding fatty acids. For microbial community analysis and relative biomass calculations, we used only fatty acids that were identifiable and present at >0.5 mol percent. The fatty acids were clustered into major microbial indicator groups, such as gram-negative bacteria (monounsaturated), gram-positive bacteria (branched-chain), and saprotrophic fungi (18:1 ω9c, 18:2 ω6c, 18:3 ω6c).

### Soil properties

We divided the cores into three categories: active, transition, and permafrost (Supplementary Figure 3). The active layer represents the surface layer of soil above permafrost that thaws and refreezes on an annual basis. The transition or transient layer typically remains frozen, but can occasionally thaw (ca. 10^1^–10^3^ yr) during warmer summers. The transition layer serves as a buffer between the active layer and permafrost by increasing the latent heat needed for thaw^64^. The boundaries of the active, transition, and permafrost layers vary in depth across basins, but general patterns are found^65^. The seasonally-thawed active layer extends from the surface soil down to the transition layer at a depth of ~34 cm. The transition layer, which episodically thaws, is typically found at a depth of 34 cm to 57 cm. Below is the permafrost that remains periannually frozen. Over time, soil in the frozen transition and permafrost layers may become progressively enriched with ice in the forms of pore ice, veins, sills, lenses, and ice wedges. The stratigraphic layers were differentiated on the basis of ice content, cryostructures, and cryofabrics.

Soil pH was measured using 1 g soil in 30 ml deionized water. Samples were shaken for 20 min and allowed to settle prior to pH measurements. Soil bulk density was calculated by dividing soil weight by soil core volume. Total soil C and N were determined by dry combustion on a Flash EA1112 elemental analyzer (CE Elantech, Inc., Lakewood, NJ). Soil C stock was calculated using the following equation: C stock (kg C·m^−2^) = % C × bulk density (g·cm^−3^) x thickness (cm) x 10^−1^. Previous determinations of soil carbon at the site^15^ showed no reaction of the soil samples with 1 *M* HCl, indicating that the soil carbon values represent SOC. Soil moisture was similar across the chronosequence, as all cores were collected frozen and the soils were saturated with water when thawed.

### Statistics

#### SSU rRNA gene amplicon analysis

To calculate beta diversities between microbial communities, the OTU table was transformed using bio-community^66^ into UniFrac format. Express Beta Diversity 1.0.4^62^ was used to calculate the unweighted unifrac beta diversity distance matrix^67^. This distance matrix was visualized using a custom program (beta diversity squares; github.com/wwood/beta_diversity_squares), which utilized a biogem^68^ built for parsing Express Beta Diversity output (bio-express_beta_diversity; github.com/wwood/bioruby-express_beta_diversity). Square sizes (side lengths) are scaled linearly according to the following formula:


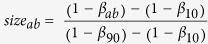


where 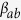 is the unweighted unifrac beta diversity between the two samples 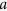 and 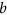, and where 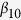 and 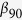 are the 10^th^ and 90^th^ percentiles respectively of all beta diversities including those between active, transition and permafrost layers.

Distances for principle coordinates analysis (PCoA) were calculated with Express Beta Diversity and PCoA performed using the cmdscale function in R and plotted using ggplot2^69^. The heatmap was created in R 3.0.1^69^ using the libraries gplots^71^ and RColorBrewer^72^.

The influence of environmental measurements on the community composition was examined using forward selection model in R. The OTU table was normalized by converting read counts to relative abundance values (treating bacteria and archaea separately), and forward selection was undertaken using the ordistep function of vegan 2.0–8^73^ with default parameters.

#### PLFA and soil properties

Microbial community lipid data and soil properties were analyzed using multivariate ordination and analysis of variance (ANOVA). Biotic and abiotic variables (microbial lipid biomass, SOC, C:N ratio, pH, bulk density, C stock, and total N) were analyzed by treatment (basin age, soil depth layer, and basin age x soil depth layer) using ANOVA. Regressions and ANOVAs of microbial lipid biomass and environmental parameters were performed with JMP 9.0 (SAS Institute Inc., Cary, NC). Statistical significance was established at the p < 0.05 level and ANOVA data were further analyzed using Tukey’s HSD *post-hoc* test.

## Additional Information

**How to cite this article**: Kao-Kniffin, J. *et al.* Archaeal and bacterial communities across a chronosequence of drained lake basins in arctic alaska. *Sci. Rep.* **5**, 18165; doi: 10.1038/srep18165 (2015).

## Supplementary Material

Supplementary Information

## Figures and Tables

**Table 1:** Classification of the basin chronosequence near Barrow, Alaska.

Category	Young	Medium	Old	Ancient
Surface organic thickness (cm)	<5	10–15	15–30	40–50
Organic form (decomposition)	fibric	fibric-hemic	hemic-fibric	sapric-hemic
Soil great groups	Aquorthel; Aquiturbel	Aquorthel; Aquiturbel	Historthel; Aquiturbel	Sapristel; Hemistel; Historthel
Preliminary ^14^C ages	<50	<320	<3000	3000–5000
Standing dead leaf material	Little standing dead except on driest areas	Moderate standing dead	Considerable standing dead especially	Much standing dead
Relative plant vigor	High	Medium to High	Moderate to Low	Low
Basin plot ID (see Fig. 1)	B3, B8, B12, N	B1, B9, B10, R1	B2, B4, B6, B11	B5, B7, R2, R3

Basin plot ID refers to the sampling points shown in Fig. 1. The criteria summarized in this table are adapted from Hinkel *et al.* (2003). Four basins were sampled at each basin age category: young, medium, old, and ancient (n = 16).

**Table 2:** Soil properties differ across the basin chronosequence and at soil depth.

	Soil pH	Bulk density	SOC	Total N	C:N Ratio	C stock (100cm)
*Basin age*		g · cm^−3^	%	%		kg · m^−2^
Young	6.8 (0.4)^a^	0.81 (0.10)^a^	6.9 (1.9)^a^	0.43 (0.11)^a^	17.9 (1.3)^a^	9.3 (2.3)^a^
Medium	6.1 (0.4)^ab^	0.58 (0.07)^ab^	8.4 (1.6)^ab^	0.49 (0.09)^a^	17.4 (1.1)^a^	10.3 (3.0)^a^
Old	5.8 (0.3)^b^	0.51 (0.08)^b^	13.4 (1.6)^b^	0.77 (0.09)^a^	17.5 (1.1)^a^	13.7 (2.9)^a^
Ancient	5.9 (0.3)^ab^	0.51 (0.07)^b^	10.7 (2.5)^ab^	0.62 (0.14)^a^	18.4 (1.1)^a^	8.5 (1.8)^a^
*Soil depth layer*						
Active (0–34 cm)	5.3 (0.2)^x^	0.59 (0.06)^x^	14. 1 (2.1)^x^	0.78 (0.11)^x^	17.6 (0.66)^x^	14.0 (2.4)^x^
Transition (34–57 cm)	6.3 (0.3)^y^	0.54 (0.10)^x^	9.6 (1.8)^xy^	0.57 (0.11)^xy^	17.6 (0.84)^x^	10.5 (2.8)^xy^
Permafrost (>57 cm)	7.0 (0.4)^z^	0.66 (0.09)^x^	5.8 (1.4)^z^	0.36 (0.09)^z^	18.3 (0.76)^x^	6.5 (1.9)^z^

The table lists the soil properties sampled across the basin successional gradient and along soil depth. The values indicate means and standard error (in parentheses). Means significantly different (p < 0.05, Tukey’s HSD) are indicated with contrasting letters. A total of 16 basins (young, medium, old, and ancient) were sampled at three soil layer depths (active, transition, permafrost).
