# Supplementary material for: Archaeal and bacterial communities across a chronosequence of drained lake basins in arctic alaska
Source: Sci Rep. 2015 Dec 18;5:18165. doi: 10.1038/srep18165 (PMC4683534; doi:10.1038/srep18165)
Supplement: Supplementary Information [file srep18165-s1.pdf]

Supplementary online material to:

Archaeal and Bacterial Communities Across a Chronosequence of Drained Lake Basins in Arctic Alaska

†J. Kao-Kniffin[1], B.J. Woodcroft[2], S.M. Carver[1], J.G. Bockheim[3], J. Handelsman[4], G.W. Tyson[2], K.M. Hinkel[5], C.W. Mueller[6]

[1] Cornell University, School of Integrative Plant Science, Ithaca, NY 14853
[2] University of Queensland, Australian Centre for Ecogenomics, School of Chemistry and Molecular Biosciences, Brisbane 4072, Queensland, Australia
[3] University of Wisconsin-Madison, Department of Soil Science, Madison, WI 53706
[4] Yale University, Department of Molecular, Cellular, and Developmental Biology, New Haven, CT 06520
[5] University of Cincinnati, Department of Geography, Cincinnati, Ohio, 45221
[6] Technische Universitaet Muenchen (TUM), Lehrstuhl fuer Bodenkunde, 85354 Freising, Germany

†Corresponding Author:
J. Kao-Kniffin
134A Plant Science Building
Cornell University
Ithaca, NY 14853
Phone: (607) 255-8886
Fax: (607) 255-0599
E-mail: jtk57@cornell.edu

Supplementary Figure Legends

Supplementary Figure 1. Drained lake basins classified by relative age. The age categories include: (a) young, (b) medium, (c) old, and (d) ancient basin. The photos represent common features of the different drained lake basins during the summer (period of annual thaw). Reprinted with permission from INSTAAR.

Supplementary Figure 2. Sampling soil cores near Barrow, Alaska. Photograph of the Big Beaver earth drill apparatus with an attached SIPRE corer measuring 150 cm long and 7.5 cm diameter. The equipment was used to sample soil cores in April, when basins were frozen. Photo taken by J. Kao-Kniffin.

Supplementary Figure 3: Zones of thaw, transition, and permafrost. Vertical profile of soil layers indicating generalized depths of active, transition, and permafrost layers. Adapted from Bockheim and Hinkel (2005).

Supplementary Figure 4: Alpha diversity in active, transition and permafrost layers. Simpson's diversity index is shown for each sample characterized, stratified by thaw layer. Transition layer samples tended to have lower alpha diversity than active or permafrost layers.

Supplementary Table 1: Sample-specific multiplex identifier (MID) barcodes used for differentiating the source of sequences output from the 454 sequencer.

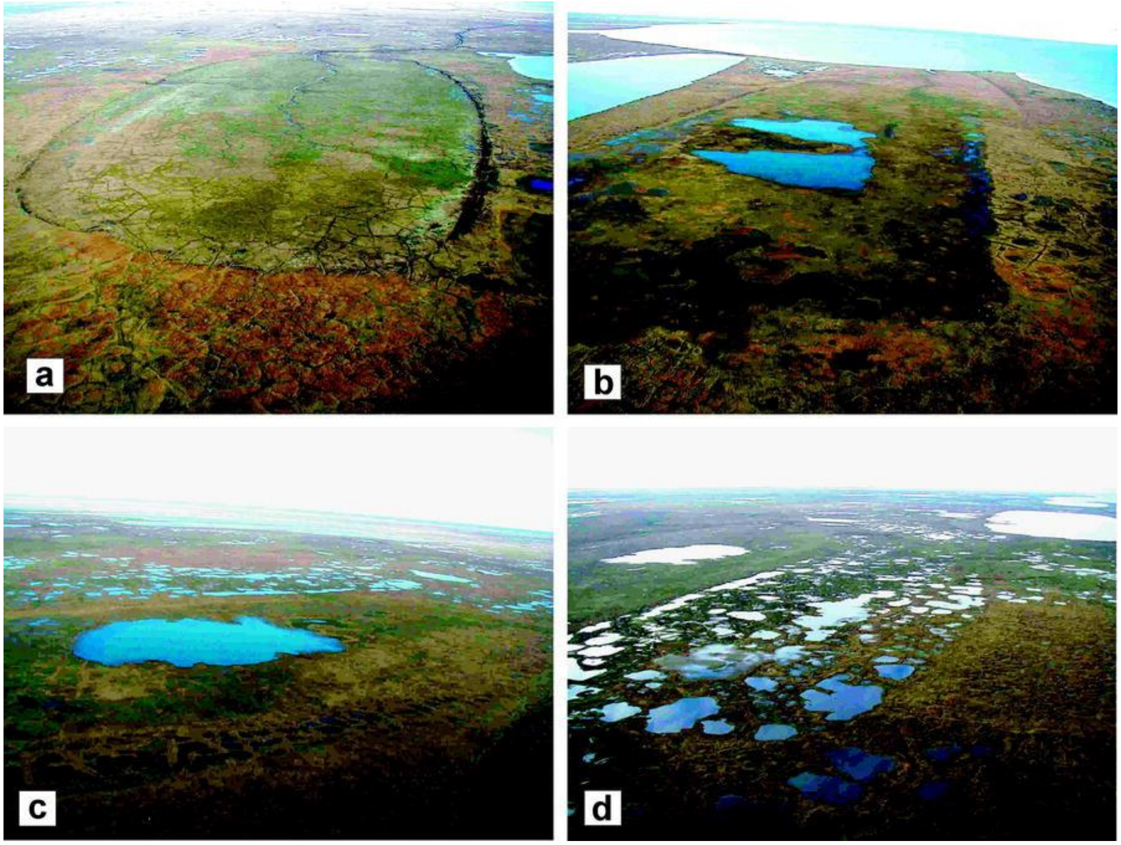

Supplementary Figure 1

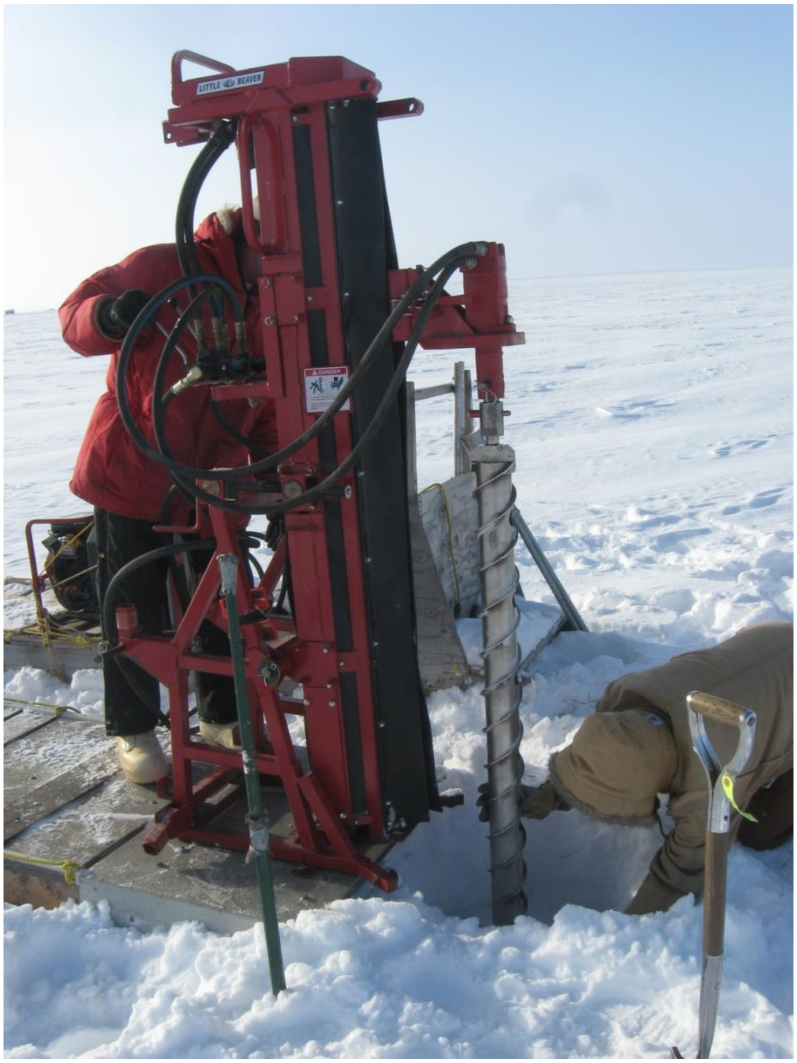

Supplementary Figure 2

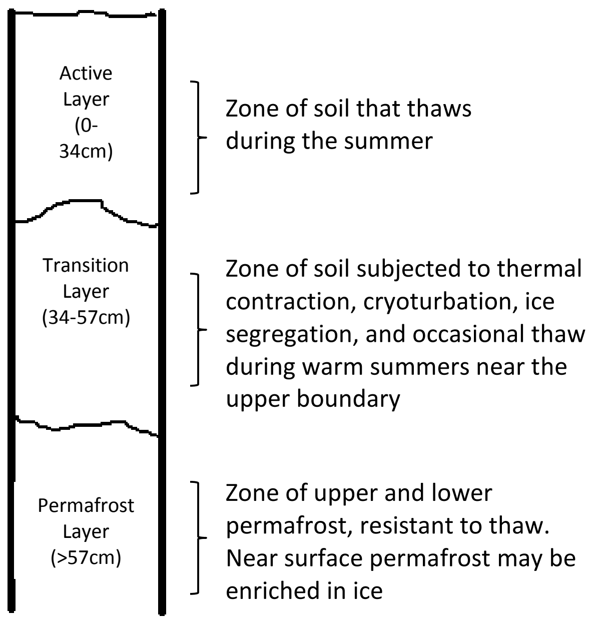

Supplementary Figure 3

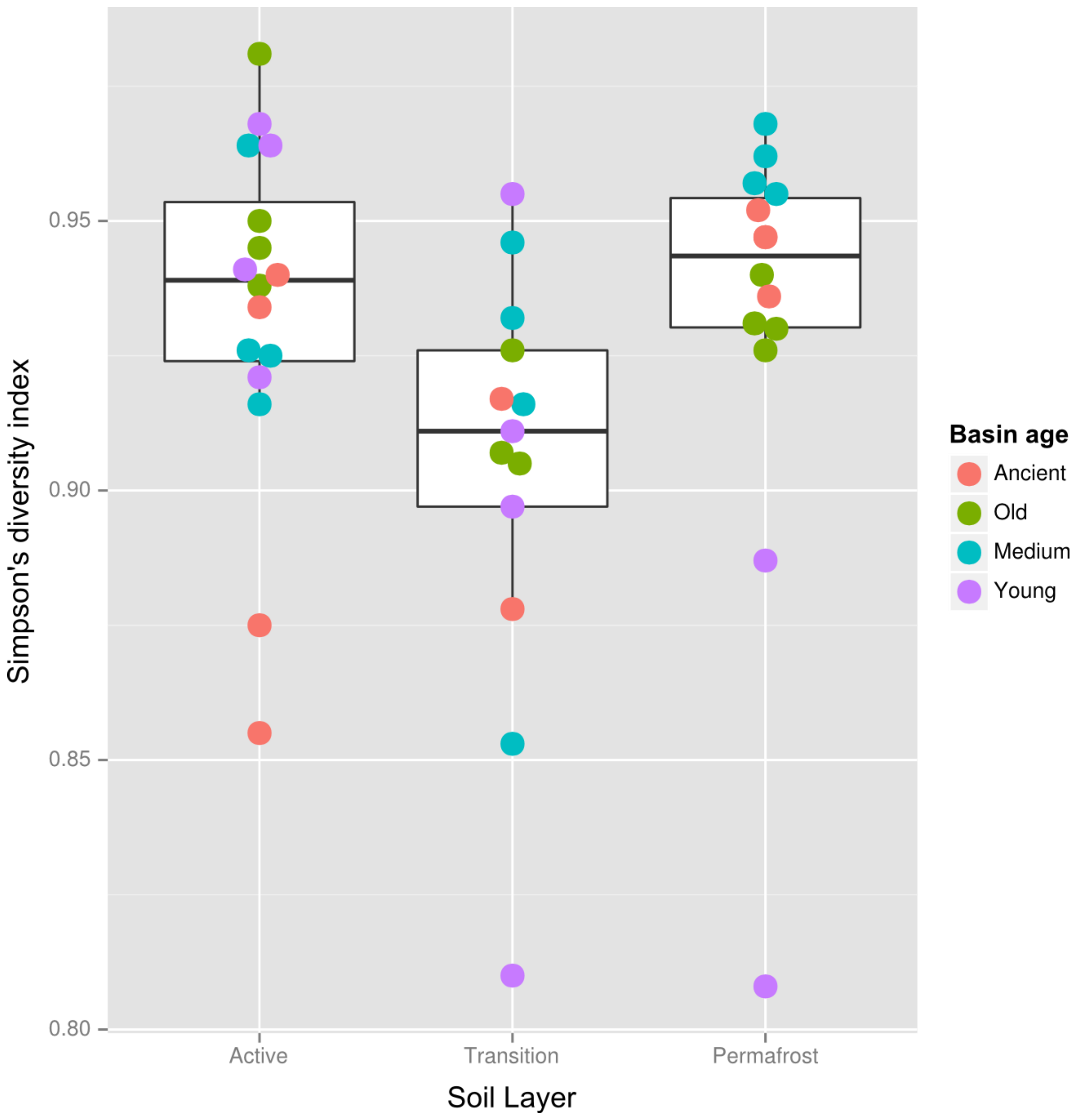

Supplementary Figure 4

Supplementary Table 1: Sample-specific multiplex identifier (MID) barcodes used for differentiating the source of sequences output from the 454 sequencer.

Depth	Basin Age	Name	MID
Active	Medium	B1	ACTAT
Active	Old	B2	TCTAC
Active	Young	B3	ACACGC
Active	Old	B4	ACAGAC
Active	Ancient	B5	ACATGT
Active	Old	B6	ACGAGT
Active	Ancient	B7	ACGCAC
Active	Young	B8	ACGTGC
Active	Medium	B9	ACTAGC
Active	Medium	B10	CACAGT
Active	Old	B11	CACGAT
Active	Medium	Rep1	CAGAGC
Active	Ancient	Rep2	CAGCAT
Active	Ancient	Rep3	CTGTAC
Active	Young	new	TACTGC
Permafrost	Medium	B1	TAGCGC
Permafrost	Old	B2	ATGCACGC
Permafrost	Old	B4	CACATCAC
Permafrost	Ancient	B5	CTATACAC
Permafrost	Old	B6	AGCAGTAC
Permafrost	Ancient	B7	AGCATAGT
Permafrost	Young	B8	ATCGACGC
Permafrost	Medium	B9	CACTGTGC
Permafrost	Medium	B10	CAGACTAC
Permafrost	Old	B11	CAGCTAGC
Permafrost	Young	B12	CAGTACGT
Permafrost	Ancient	Rep3	CTATGAGT
Transition	Medium	B1	TAGCTCAT
Transition	Old	B2	TATGATAT
Transition	Young	B3	TCAGCTAC
Transition	Old	B4	TCTCGAGT
Transition	Ancient	B5	TGCGCGAT
Transition	Young	B8	ACAGTGTGT
Transition	Medium	B9	ACATATAGT
Transition	Medium	B10	ACGAGACAC
Transition	Old	B11	ACGCAGCGC
Transition	Young	B12	ACGCGATGT
Transition	Medium	Rep1	ACGTCTCAT

Transition	Ancient	Rep2	ACTGAGCAT
Transition	Young	New	AGACACTGT
Active	Young	B12	CGTCAGCA
Permafrost	Medium	Rep1	CTAGATCA
Permafrost	Ancient	Rep2	CTGTGTGC
